# Obesity, Cardiorespiratory Fitness, and Cardiovascular Disease

**DOI:** 10.1007/s11886-023-01975-7

**Published:** 2023-10-13

**Authors:** Amier Haidar, Tamara Horwich

**Affiliations:** Division of Cardiology, David Geffen School of Medicine at UCLA, Los Angeles, CA USA

**Keywords:** Obesity, Cardiorespiratory fitness, Cardiovascular disease

## Abstract

**Purpose of Review:**

Obesity, generally defined by body mass index (BMI), is an established risk factor for the development of cardiovascular disease (CVD), while cardiorespiratory fitness (CRF) decreases risk. In chronic CVD, an obesity survival paradox in which higher BMI is associated with improved prognosis has been reported. This paper will examine the effect of obesity on CVD risk, explore obesity as a risk factor in patients with established CVD, and investigate the relationship between CRF, obesity, and CVD.

**Recent Findings:**

Through metabolic and hemodynamic changes, obesity increases the risk for CVD and contributes to the development of other cardiovascular risk factors such as diabetes, dyslipidemia, and hypertension. Obesity is associated with metabolic, hormonal, and inflammatory changes that leads to atherosclerosis increasing the risk for coronary artery disease, and myocardial remodeling increasing the risk for heart failure. However, it has also been observed that overweight/obese patients with established CVD have a better prognosis when compared to non-obese individuals termed the obesity paradox. CRF is a vital component of health associated with improved cardiovascular outcomes and furthermore has been shown to markedly attenuate or nullify the relationship between obesity and CVD risk/prognosis.

**Summary:**

Increasing CRF mitigates CVD risk factors and improves overall prognosis in CVD regardless of obesity status.

## Introduction

Obesity rates have been increasing in the USA over the past decades and are projected to continue to rise with an estimation that, by 2030, 78% of American adults will be overweight or obese (body mass index [BMI] ≥ 25 kg/m^2^), including 50% of adults having obesity (BMI ≥ 30 kg/m^2^) [1•, 2–5]. It is projected that the largest increases in obesity rates will be observed in women, low-income adults, and Hispanic and Black populations [2, 4, 5]. Since obesity is an established risk factor for cardiovascular disease (CVD) [6•, 7–9] and contributes to diabetes, dyslipidemia, and hypertension [3, 6•, 9], a rise in obesity may contribute to cardiovascular health disparities in the USA.

Obesity as a disease is heterogeneous and multi-faceted, with a myriad of potential influences and causes. Lifestyle, socioeconomic, and environmental factors all contribute to obesity [10]. Obesity is traditionally defined by BMI, although a call for alternative methodology to define obesity is rising. Lifestyle factors that have been associated with obesity include diet, physical activity, sleep, and alcohol consumption [10]. Living in poverty, unemployment, housing insecurity, education, and social support are socioeconomic factors that contribute to obesity [10]. Lastly, environmental factors such as food insecurity, access to supermarkets, state and local policies, marketing, and access all are potential influences for the development of obesity [10]. Together, these factors not only influence the likelihood of developing obesity but also may contribute to the overall cardiorespiratory fitness (CRF) of individuals [11].

Cardiorespiratory fitness (CRF) refers to the ability of the circulatory and respiratory systems to supply oxygen to skeletal muscle during prolonged moderate-to-vigorous exercise [11–13]. Clinically, CRF may be reported as Vo_2_max (mlO2·kg^−1^·min^−1^) measured using treadmill or bicycle ergometer exercise tests or reported as metabolic equivalents (METs) [12, 13]. Poor CRF has been associated with increased risk for developing CVD risk factors, including metabolic syndrome, hypertension, dyslipidemia, diabetes, and atrial fibrillation [14–16]. Low CRF is also a predictor of cardiovascular disease development, all-cause mortality, CVD-related mortality, and cancer-related mortality [17••, 18–21]. Despite the importance of CRF on health and long-term mortality, the CRF of adults in the USA and internationally has decreased over the past 40 years, with the largest declines coinciding with the largest increases in adult obesity [22]. In this review, we will discuss obesity and CVD risk, obesity as a risk factor in patients with established CVD, and the relationship between CRF, obesity, and CVD.

## Obesity and Cardiovascular Disease Risk

A host of pathophysiologic cardiovascular changes occurs in response to excess body mass in obesity [8]. The cardiac remodeling that occurs is multi-factorial and involves a combination of changes in hemodynamics, neurohormonal signaling, and/or myocardial metabolism [23]. Metabolically, obesity induces myocardial cellular mechanisms such as ectopic cardiac fat deposition, toxic lipid metabolite accumulation, mitochondrial dysfunction, and inflammation that lead to metabolic adaptations [23]. Hemodynamically, the metabolic demand of obesity is associated an increase in intravascular blood volume and cardiac output through an increase in stroke volume and heart rate [8]. This increased workload predisposes the heart to structural remodeling including concentric and eccentric left ventricular hypertrophy [8].

It has been observed that a subset of individuals, despite having obesity, has a normal cardiometabolic profile [24]. Termed metabolically healthy obesity (MHO), it is defined as having a BMI > 30 kg/m^2^ and meeting none of the metabolic syndrome criteria except waist circumference [24]. Initially, it was thought that MHO individuals had similar CVD risk as compared to normal weight metabolically healthy individuals [25]. However, recent studies have shown that MHO is not a benign condition, and any level of obesity increases the risk for cardiovascular disease [8, 26•, 27–29]. MHO has been associated with coronary artery calcification, impaired vasoreactivity and left ventricular function, and increased carotid intima-media thickness [8, 30–33]. Individuals with MHO are also more quickly and likely to develop metabolic syndrome and have a higher risk of coronary artery disease, heart failure, and all-cause mortality when compared to metabolically healthy, non-obese individuals [34–37]. Thus, the metabolic and hemodynamic changes that occur in obesity, even in the absence of traditional metabolic risk, increase the risk for both coronary artery disease (CAD) and heart failure, as well as arrhythmias such as atrial fibrillation.

### Obesity and Coronary Artery Disease

Obesity is an independent risk factor for CAD and contributes to other risk factors for the development of CAD including insulin resistance, hypertension, and dyslipidemia [6•, 9, 26•, 29, 38]. Obesity leads to CAD in part through an inflammatory process that accelerates atherosclerotic plaque formation which may start as early as childhood [6•, 7]. This inflammatory state promotes the oxidation of low-density lipoproteins (LDL) which promotes atherogenesis [7]. Additionally, inflammation and oxidative stress diminishes the availability of nitric oxide leading to endothelial dysfunction [39]. Adipose tissue’s role as an endocrine organ contributes to the relationship between obesity and CAD, which is the role of adipose tissue as an endocrine organ. Adipose tissue has the ability to release adipokines which are bioactive molecules consisting of hormones, chemokines, and cytokines [8, 40]. Adipose tissue dysfunction in obesity leads to a proliferation of pro-inflammatory adipokines [8, 41] which promotes atherosclerosis by inducing insulin resistance, endothelial dysfunction, hypercoagulability, and systemic inflammation [7].

### Obesity and Heart Failure

Obesity is a significant risk factor for heart failure with both preserved and reduced ejection fraction [6•, 42]. Obesity leads to heart failure through hemodynamic and metabolic changes that affect the myocardium [7, 9, 23]. Both the renin-angiotensin-aldosterone system and the sympathetic nervous system are upregulated in obesity [7]. Associated metabolic, hormonal, and inflammatory changes in obesity contribute to myocardial remodeling, increasing the risk for heart failure [23]. The obesity-related pro-inflammatory environment promotes atherosclerosis which also may eventually lead to systolic dysfunction and clinical heart failure [9]. Obesity also leads to the deposition of adipokine-releasing epicardial fat [23]. Fat deposition within and around the heart promotes cardiac dysfunction and subsequent heart failure, through metabolic alterations, lipotoxicity, and myocardial fibrosis [23, 43–46]. In summary, the pathophysiology of obesity in heart failure is complex and involves several direct and indirect mechanisms, all of which lead to metabolic, structural, and functional remodeling and ultimately systolic or diastolic heart failure.

## The Obesity Paradox

While obesity is associated with a number of established CVD risk factors, it has also been observed that overweight/obese patients with established CVD have a better prognosis when compared to non-obese individuals in some specific populations [9, 24, 27, 29, 47]. This obesity paradox has been observed in several conditions and types of CVD including heart failure, coronary artery disease, and atrial fibrillation (Table 1) [8, 9, 24, 29].

**Table 1:** Conditions associated with an obesity paradox

Coronary heart disease
Heart failure
Hypertension
Pulmonary arterial hypertension
Atrial fibrillation
Chronic obstructive pulmonary disease
End-stage renal disease
Rheumatoid arthritis
Various cancers
HIV

There are several possible explanations for this obesity survival paradox. Arguments for the obesity paradox include greater metabolic reserve in the setting of the catabolic state of heart failure, protective alterations in cytokines and adipokines, favorable hemodynamic profile due to low levels of circulating B-type natriuretic peptide, anti-inflammatory effects from elevated circulating lipoproteins, attenuated response to renin-angiotensin-aldosterone system activation, the potential for fat as a repository for stem cells to repopulate the myocardium in heart failure, heightened symptoms in obesity leading to earlier presentation, and the ability to tolerate higher doses of cardioprotective blood pressure lowering medications [8, 47]. Others contend that the concept of an obesity paradox may be flawed due to multiple biases and confounders influencing the association between obesity and CVD, the use of BMI as the main measure of obesity rather than accounting for body composition and adipose distribution, and the disappearance of the paradox in individuals with higher cardiorespiratory fitness (CRF) [8, 47, 48].

Overall, despite the numerous studies and analyses that have demonstrated an obesity paradox in patients with CVD, an improved prognosis has been consistently seen in association with increased physical activity, exercise training, and higher levels of cardiorespiratory fitness [24, 27, 38, 49]. Furthermore, CRF has been shown to possibly confound the relationship between obesity and CVD risk. MHO individuals have been shown to have higher levels of CRF when compared to metabolically abnormal obese individuals, with higher fitness levels associated with a reduction in CVD risk [50].

## Cardiorespiratory Fitness

Although it has been established that low CRF is associated with a higher risk of CVD and all-cause mortality [13–16, 17••, 18, 20], CRF among adults has been on the decline over the past 40 years [22]. In one of the largest meta-analyses to date, CRF was inversely associated with all-cause mortality, with every 1 metabolic equivalent increase in estimated CRF (eCRF) associated with an 11% reduction in mortality [51, 52]. This inverse relationship has been demonstrated to exist in individuals with and without CVD [53, 54]. Despite this, CRF is not routinely or regularly assessed by physicians as part of an overall health assessment [13, 55, 56]. CRF can be quantified using measures obtained from cardiorespiratory exercise testing (CPX) which provides peak oxygen consumption (Vo_2_peak) and other parameters, including the minute ventilation/carbon dioxide production (Ve/ Vco_2_) slope or exercise testing without CPX measurements [13]. Non-exercise measures of CRF include various models that incorporate clinical health indicators, which may be useful in estimating initial CRF [13, 57]. Determinants of CRF, in healthy individuals, include age, BMI, waist circumference, body fat, weight, smoking, and physical activity levels [12].

**Fig. 1:**
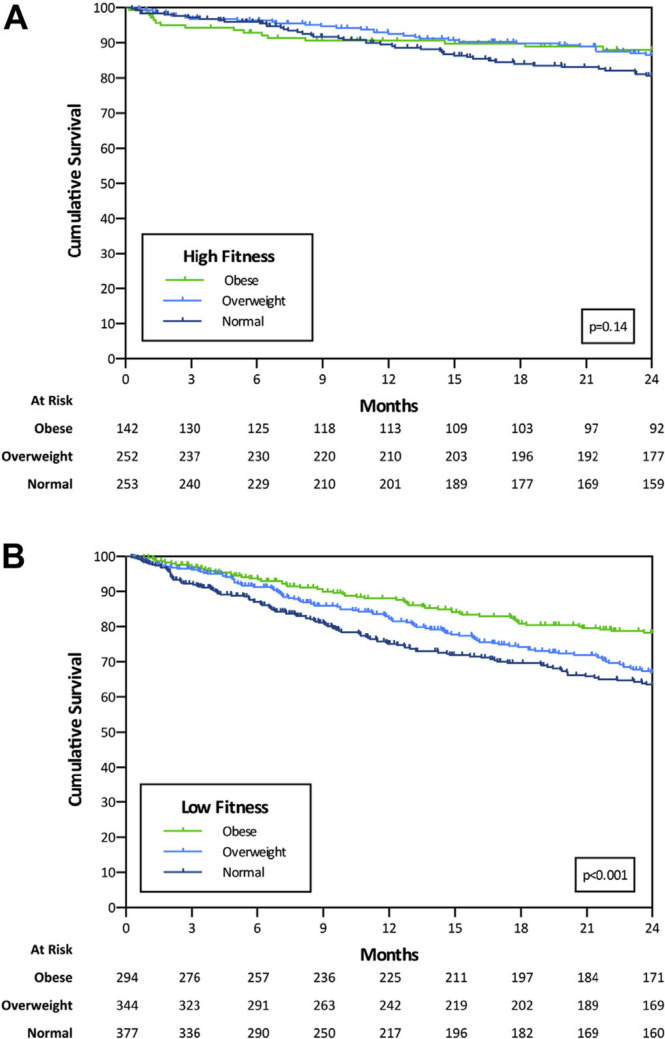
Survival at 2 years by BMI in PKVO2 subgroups: **A** high PKVO2 and **B** low PKVO2 (adapted from Clark et al. Am J Cardiol 2015;115(2):209–213, with permission from Elsevier) [60]

There has been consistent evidence of an association between BMI, waist circumference, and CRF [12]; higher levels of obesity are associated with lower levels of CRF [58]. However, individuals with obesity can have varying levels of CRF [59]. Together, levels of obesity and CRF interact to determine an individual’s CVD risk and CVD prognosis [60]. CRF can mitigate obesity-related CVD risk, as studies have shown an improvement in outcomes among obese individuals with higher levels of CRF [18, 27, 29, 61]. In men with known or suspected coronary heart disease, CRF modified the relationship of adiposity to mortality, with no significant differences in CVD and all-cause mortality risk across BMI, WC, and percent BF categories among men with high fitness levels [62]. Low CRF has been associated with increased risk for heart failure across all BMI categories, with differences in CRF levels serving as an underlying reason for the association between obesity and heart failure [63]. The effect of CRF on CVD prognosis regardless of BMI has been termed the “fat but fit” phenomenon [29, 61]. In patients with systolic heart failure, one study demonstrated an obesity paradox only in those with low CRF, revealing increased mortality in non-obese patients (Fig. 1) [64]. This association between BMI and mortality disappeared in the high CRF group. When BMI and CRF were examined together, individuals with high CRF had the best survival outcomes regardless of BMI, suggesting that improved fitness may attenuate the obesity paradox. While various studies have examined the relationship between obesity, CRF, and CVD, it is important note that obesity is still a significant risk factor for the development of CVD [38]. Overall, fitness and adiposity interact to determine CVD risk, and weight loss when combined with improvements in fitness may lead to the best prognosis in individuals with obesity and CVD [38, 65].

Increased efforts are needed to improve CRF in individuals with CVD. Cardiac rehabilitation and physical activity interventions have been shown to improve CRF in patients at risk for CVD [8, 12, 13]. Among patients with established CVD, exercise training improves CRF leading to improved prognosis, outcomes, and overall quality of life [49, 66]. Various types of physical activity increase CRF in most adults, with the greater the amount or intensity of physical activity, the greater the increase in CRF [13]. The relationship between physical activity, CRF, obesity, and CVD is complex; however, it is well documented that participating in physical activity increases and allows for the highest possible level of CRF, which, in turn, mitigates CVD risk factors and improves overall prognosis of both CAD and HF [12, 28, 57].

## Conclusion

In conclusion, obesity is a significant risk factor for CVD and contributes to the development of other cardiovascular risk factors such as diabetes, dyslipidemia, and hypertension. Obesity-associated changes in hemodynamics, neurohormonal signaling, and myocardial metabolism increase the risk for both CAD and HF. While obesity increases the risk for CVD, it has been observed that overweight/obese patients with established CVD such as heart failure and CAD have a better prognosis when compared to non-obese individuals. While there are many explanations for this paradox, CRF has been shown to markedly attenuate the relationship between obesity/elevated BMI and CVD risk/prognosis. Participating in physical activity increases and allows for the highest possible level of CRF, which is associated with lowered risk of CVD incidence and mortality. Taken together these data suggests that fitness may surpass fatness in determining long-term CVD prognosis.
